# Range-weighted branch length difference (RWiBaLD), a new method for distinguishing meso-endemism from neo-endemism and paleo-endemism

**DOI:** 10.1371/journal.pone.0349623

**Published:** 2026-05-22

**Authors:** Brent D. Mishler, Nunzio Knerr, Joseph T. Miller, Andrew H. Thornhill, Shawn W. Laffan

**Affiliations:** 1 Department of Integrative Biology, University and Jepson Herbaria, University of California, Berkeley, California, United States of America; 2 CSIRO National Research Collections Australia, Australian National Herbarium, Canberra, Australian Capital Territory, Australia; 3 Global Biodiversity Information Facility, Copenhagen, Denmark; 4 N.C.W. Beadle Herbarium, School of Environmental and Rural Science - Botany, University of New England, Armidale, New South Wales, Australia; 5 Earth and Sustainability Science Research Centre, School of Biological Earth and Environmental Sciences, University of New South Wales, Sydney, Australia; University of Lodz Faculty of Biology and Environmental Protection: Uniwersytet Lodzki Wydzial Biologii i Ochrony Srodowiska, POLAND

## Abstract

Our goal is to enhance understanding of phylogenetic endemism (PE), which is studied using a range-weighted phylogenetic tree. We present a new method called ***R****ange* ***W****e****i****ghted* ***B****r****a****nch* ***L****ength* ***D****ifference* (RWiBaLD), which is based on the difference between the length of a branch on a range-weighted observed tree and its length on the corresponding range-weighted comparison tree. The latter is a tree with the same topology as the observed tree but with branches adjusted to be of equal length before range-weighting. The goal of this method is to detect the branches of the phylogeny that contribute most to PE, and to distinguish among neo-endemism, paleo-endemism, and a novel category: meso-endemism. The latter is a heretofore missing category in the study of endemism referring to a highly range-restricted lineage that is not particularly short (neo-) or long (paleo-). Application of this approach is illustrated using the plant genus *Acacia* across the continent of Australia, using previously published spatial data and phylogeny. We show the properties of RWiBaLD, and how to apply it by: (1) mapping the outputs on a phylogeny, thus for the first time enabling PE to be scored for use in existing phylogenetic comparative methods, and (2) dissecting the composition of concentrations of PE on the landscape that are detected by the *Categorical Analysis of Neo- And Paleo-Endemism* (CANAPE) method. Whereas CANAPE identifies geographic concentrations of high PE, and gives a summary classification of the type of endemism dominating in a location, RWiBaLD identifies the specific branches that contribute the most to PE, and what type of endemism they represent. Thus, RWiBaLD enables novel studies of the evolutionary and ecological causes of endemism, as well as improved conservation assessment.

## Introduction

Spatial patterns of biodiversity are commonly measured by examining changes in species distributions across a region to identify areas of particularly high diversity and endemism. Beta-diversity, or biotic turnover on the landscape, is likewise usually measured by comparing proportions of species shared among areas. However, investigations based on species distributions alone miss the full richness of analyses that can result from taking a phylogenetic approach. Spatial phylogenetics is a field that uses a phylogeny integrated with spatial data to assess biodiversity in an evolutionary context using explicit statistical tests [1–12].

Documenting patterns of phylogenetic diversity and endemism on the landscape is important for understanding evolutionary and ecological processes affecting biodiversity. It is also important for conservation planning, given the need to prioritize efforts in the face of rapid habitat loss and human-induced climate change [13]. These spatial phylogenetic methods allow assessments of protected lands that are not limited by reliance on species distribution alone and can identify complementary areas of biodiversity that have unique evolutionary histories in need of conservation [14].

Spatial phylogenetics utilizes a range of diversity metrics. Phylogenetic diversity (PD), first described by Faith [15], is the sum of the branch lengths connecting the taxa that are present in a particular location to the base of the tree. It can be expressed in absolute tree units or as a relative proportion of the tree’s overall length (the sum of its branch lengths). Phylogenetic endemism (PE) [16] is a range-weighted variant of PD where the phylogenetic branch lengths are weighted by the fraction of their geographic range represented by the sample area [17,18]. In this way, narrow-ranged branches contribute more of their length to the PE score, while wide-ranged branches contribute less. The ranges of internal branches are calculated as the union of their descendent terminals.

Relative Phylogenetic Diversity (RPD) and Relative Phylogenetic Endemism (RPE) are related metrics; both are ratios that respectively compare PD or PE measured on an observed phylogeny against PD or PE measured on a comparison phylogeny that has the same topology but with all branches adjusted to be of equal length [1]. RPD and RPE are designed to detect and quantify statistically significant concentrations of unusually short or long branches. These phylodiversity metrics are all rank-free since it does not matter what taxonomic levels the terminals represent, provided they are monophyletic, and their geographic distributions can be characterized. Furthermore, deciding whether one species is really two species is not nearly as consequential to branch length calculations as it is to methods using species richness as a metric.

### Range-weighted trees and CANAPE

The most common applications of PE use equal-area spatial units, typically square grid cells, where each has a weight of 1, and the PE score for each spatial unit is calculated in isolation, not considering collections of neighboring units [19]. In such cases the PE weighting can be described as using the inverse of the branch range. Given that this weighting is the same across all grid cells it is possible to create a *range weighted tree* (RWT) with the same topology as the original tree but with the original branch lengths each divided by their range sizes. The RWT is extremely useful in several contexts, including as the basis for PE: PE can be most simply understood as PD measured on a RWT. Another use of the RWT is in the quantification of biotic turnover as range-weighted phylogenetic turnover [18], which is useful for biotic regionalization as well as in complementarity analyses for applied conservation planning studies [14].

An additional use of range-weighted trees is in the RPE metric [1]. The standard PE measure uses the range-weighted observed tree (RWoT, the numerator in RPE), which confounds observed branch length and range size and thus emphasizes paleo-endemism (i.e., range-restricted long branches). The denominator in RPE uses the range-weighted comparison tree, a tree with all branches set to the same length (i.e., an equal proportion of the total tree length) before range-weighting (RWcT), This enables a level playing field where neo-endemism (range-restricted short branches) can be studied on a equal basis with paleo-endemism.

Categorical Analysis of Neo- And Paleo-Endemism (CANAPE) is a two-step method that employs PE, RPE, and a spatial randomization to allow a clear, quantitative distinction between centers of neo- and paleo-endemism across a region [1]. The randomization of terminal taxon occurrences has two constraints: richness of each grid cell remains constant and number of grid cells (range size) of each terminal remains constant. Step one of CANAPE is to identify locations that have significantly high PE (one-tailed test) on either or both of the RWoT or the RWcT. Step two is to examine the significance of the RPE ratio itself (two-tailed test) for those locations. A significantly high ratio means the range-restricted branches in that location are longer than expected, indicating a concentration of paleo-endemism. A significantly low ratio means the range-restricted branches in that locality are shorter than expected, indicating a concentration of neo-endemism. Locations with a non-significant ratio in step 2 were described [1] as concentrations of “mixed-endemism” or “super-endemism” (with the latter term applied if either PE score in step 1 is highly significant); the interpretation being that the location contains some mixture of range-restricted branch lengths but is not dominated by either neo-endemism or paleo-endemism.

### Range weighted branch length difference (RWIBALD)

While CANAPE is effective in generating a summary classification for locations, it was not meant to give details about what was happening within locations. In particular, areas classified as containing “mixed endemism” are likely to be heterogeneous. We realized that a method was needed to drill into each location to examine the contribution of individual branches of the phylogeny to patterns of PE, thereby addressing questions such as: Which particular range-restricted long branches are present in an area of significantly high PE? What ecological traits are associated with branches showing neo-endemism? Furthermore,it remains to be explored whether range-restricted branches that are neither particularly long or short are contributing significantly to patterns of endemism.

Here we present a new method called ***R****ange* ***W****e****i****ghted* ***B****r****a****nch* ***L****ength* ***D****ifference* (RWiBaLD), based on the difference between a branch’s length on the RWoT and on the RWcT, and used to identify specific phylogenetic branches that are contributing neo-endemism, paleo-endemism, or a newly proposed category: *meso-endemism*. The latter is a heretofore missing category in the study of endemism referring to range-restricted branches of intermediate length; this category is intermediate on a continuum between neo-endemism (range-restricted branches of shorter than expected length) and paleo-endemism (range-restricted branches of longer than expected length). Such branches may be important contributors to endemism but have been ignored by previous methods focused solely on distinguishing neo- and paleo-endemism.

**Fig 1:**
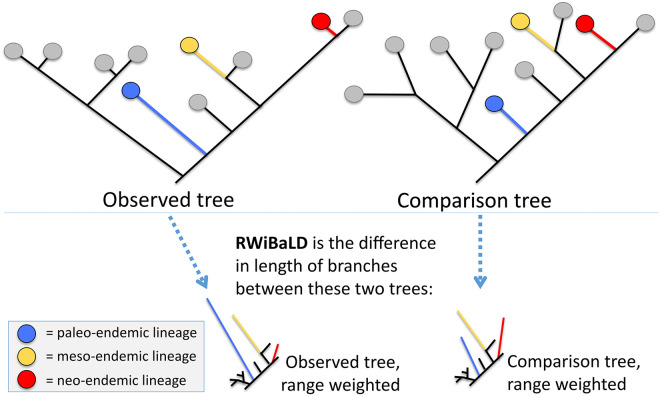
A simple example illustrating RWiBaLD, using a hypothetical phylogeny showing the observed branch lengths estimated from the data (these also can be time-calibrated branch lengths), a comparison tree that retains the same topology but has all branch lengths adjusted to be of equal length, and the range-weighted (RW) version of both. The RWiBaLD score is the difference between the two RW trees for a given branch’s length. In this example only three of the terminal branches (shown in color) are highly range-restricted. Note that the blue branch (representing a potential paleoendemic) is much longer on the RWoT than the RWcT, the red branch (representing a potential neoendemic) is much shorter on the RWoT than the RWcT, and the yellow branch (representing a potential mesoendemic) is about the same length on the RWoT and the RWcT.

CANAPE is best viewed as a way of classifying *locations*; it provides a summary of the overall distribution of range-restricted branches in a location, and their significance across the map. But, as with any summary measure, there is considerable variation concealed within its categories. RWiBaLD is best viewed as a method of classifying *branches*. When we refer to a “branch” in this paper we are referring specifically to a *lineage segment*, which is the interval along a lineage between two branching events, or between one branching event and the present [20]. RWiBaLD scores can be applied to all branches as a continuous variable; we also present an approach for applying it as a useful categorical variable that focuses on those range-restricted branches that contribute the most to PE and partitions them into three categories: highly negative (neo-endemic branches), highly positive (paleo-endemic branches), and intermediate (meso-endemic branches). See Fig 1 for a simple diagram illustrating these categories.

**Fig 2:**
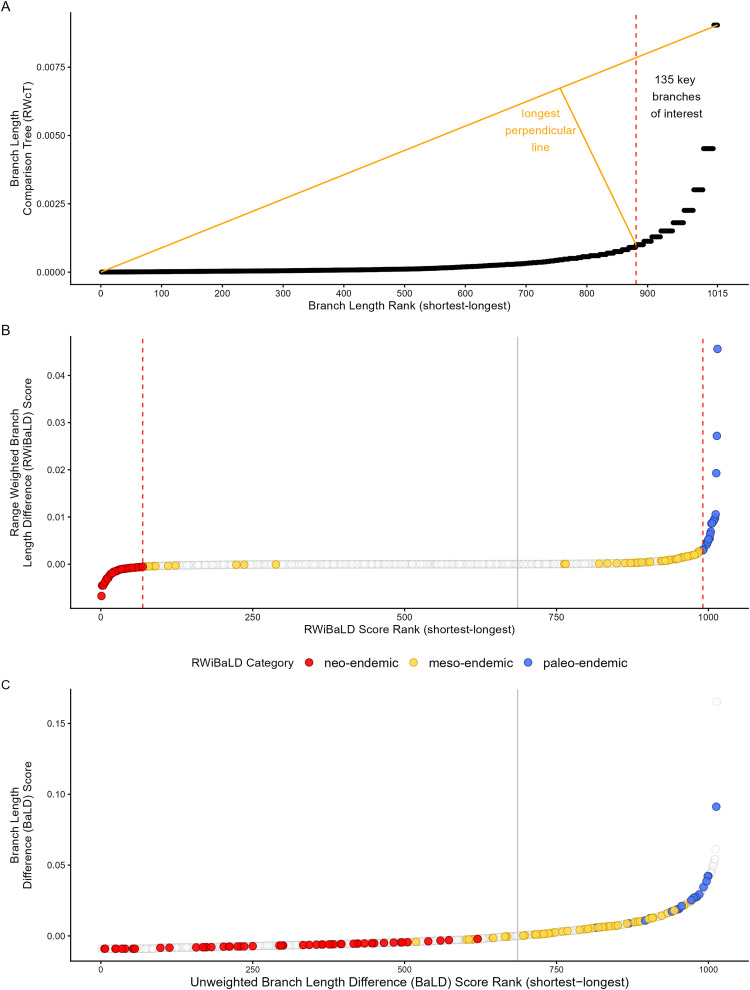
An illustration of the RWiBaLD protocol, as applied to the Australian *Acacia* data. **A**. Illustrating the choice of branches of interest based on their contribution to PE; the Y-axis is the branch length on the range-weighted comparison tree (inversely proportional to the range size of the branch); the X-axis is all branches ordered by range size. The dotted red line indicates the cut-off based on the elbow method (orange lines indicate the cut-off calculation: drawing a line between the furthest points, then finding the longest perpendicular line that meets the curve). **B**. Illustrating the choice of thresholds for defining 3 categories of endemism; the Y-axis is the RWiBaLD score for each branch; the X-axis is all branches ordered by RWiBaLD score. The dotted red lines indicate the cut-offs for each half of the distribution, based on the same elbow method applied separately to each half. The branches of interest are colored by category; the remainder of branches are uncolored. The grey line indicates where RWiBaLD = 0. **C.** Illustrating the differences between branch lengths on the observed tree and the comparison tree *with no range-weighting* of either; the Y-axis is the difference between length on the observed tree and length on the comparison tree for each branch (referred to as BaLD); the X-axis is all branches ordered by their BaLD score. The branches of interest are colored by RWiBaLD category as defined in Fig 2B, and the grey line indicates where BaLD = 0.

RWiBaLD works using the following protocol; an implementation using the R language and *Biodiverse* software [21] is given as a quarto document, PDF, and as HTML (https://github.com/NunzioKnerr/rwibald):

1. For each branch on the phylogeny (terminal or internal), calculate its *range weighted branch length difference* (RWiBaLD) score as the difference between its length on the RWoT and the RWcT. These scores can be used directly as a continuous measure of neo- and paleo-endemism (negative and positive values, respectively). Classification into neo-endemic, paleo-endemic, and meso-endemic requires further processing using steps 2–4:
2. Identify the “highly endemic branches,” i.e., those with range sizes below a threshold. We calculate this threshold by identifying the “elbow” of the distribution, using the *maximum Euclidean distance* method [22]. This is done by ranking all branches according to the inverse of their range size (functionally the same as their lengths on the RWcT), plotting a straight line from the first to last points on that curve, then identifying the point on the curve that produces the longest perpendicular line drawn to it from the straight line (Fig 2A). Branches greater than or equal to the threshold are considered the highly endemic branches of interest. This method provides a data-driven and reproducible way to define a threshold for highly endemic branches without imposing arbitrary cutoffs. Using maximum Euclidean distance to identify the point of maximum curvature in the ranked distribution of inverse range sizes isolates branches whose geographic restriction is exceptional relative to the full distribution, rather than based on absolute range size alone. The resulting threshold adapts naturally to differences in phylogenetic structure and spatial sampling across datasets, and branches with inverse range sizes greater than or equal to this value are classified as highly endemic and retained for subsequent neo-, meso-, and paleo-endemism classification.
3. The same elbow statistic is then applied to the RWiBaLD scores from step 1. The positive and negative differences are processed separately (divided at the RWiBaLD = 0 value), resulting in two thresholds (Fig 2B).
4. The highly endemic branches identified in step 2 are then classified using the thresholds from step 3. Highly endemic branches with negative differences less than or equal to the threshold are classified as neo-endemic, those with positive differences greater than or equal to the threshold are classified as paleo-endemic, and those between these two categories are classified as meso-endemic (Fig 2B).

## Case study

To examine the properties of RWiBaLD and to illustrate its application with real data, we used the phylogeny and spatial dataset of the Australian angiosperm genus *Acacia* as published [1]. The spatial dataset is available from Dryad (http://doi.org/10.5061/dryad.dv4qk) and the phylogenetic dataset and tree are available from TreeBase (https://treebase.org/treebase-web/search/study/summary.html?id=13659). We calculated the RWiBaLD distribution following the protocol above.

We found 135 highly endemic branches (protocol step 2; Fig 2A). Using steps 2 and 3, these can be divided into 55 neo-endemic branches, 59 meso-endemic branches, and 21 paleo-endemic branches (Fig 2B). The RWiBaLD scores and categories for each branch are provided in the output file “Acacia_RWiBaLD_results_all_with_range.csv” in the quarto outputs folder (https://github.com/NunzioKnerr/rwibald/quarto_outputs/Acacia_RWiBaLD_results_all_with_range.csv). *Note that these RWiBaLD results are properties of the branches*, i.e., discrete or continuous traits that can be studied using standard phylogenetic comparative methods, a novel contribution of the RWiBaLD method.

**Fig 3:**
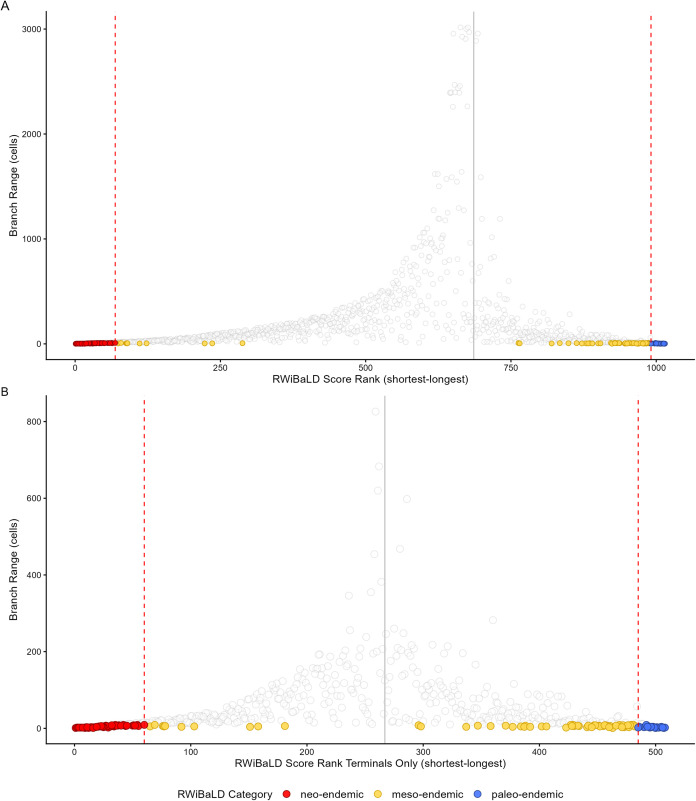
The distribution of RWiBaLD results on the *Acacia* phylogeny. **A.** graph showing rank order of RWiBaLD scores (X-axis) relative to range size (Y-axis) for *all branches* on the tree. **B.** graph showing rank order of RWiBaLD scores (X-axis) relative to range size (Y-axis) for *only the terminal branches* on the tree. In both, the branches of interest are colored by RWiBaLD category and the dotted red lines indicate the cut-offs for each half of the distribution, from Fig 2B. The grey line indicates where RWiBaLD = 0.

For comparison with RWiBaLD we also computed a similar metric that shows the differences between branch lengths in the observed and comparison trees *with no range-weighting* (which we simply call **B**r**a**nch **L**ength **D**ifference, or BaLD), as shown in Fig 2C. The distribution of RWiBaLD scores in relation to the range sizes of branches was visualized in two ways (Fig 3), one including all branches and one including only terminal branches.

**Fig 4:**
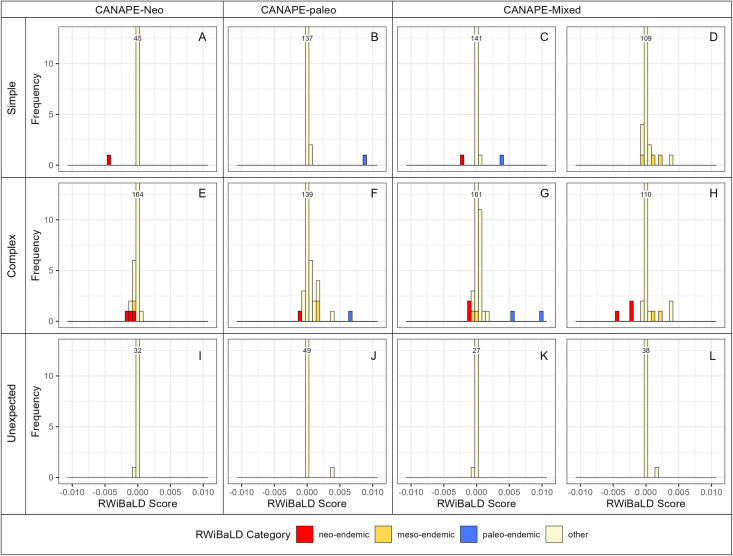
Histograms of RWiBaLD scores of branches from the *Acacia* phylogeny for 12 selected grid cells that were significant in CANAPE in Mishler (2014). The first column are cells that were classified by CANAPE as centers of neo-endemism, the second column are cells that were classified as centers of paleo-endemism, and the third and fourth columns are cells that were classified as centers of mixed-endemism. Neo-endemic branches are shown in red, mesoendemic branches in yellow, and paleoendemic branches in blue. The top row shows grid cells selected to illustrate patterns expected from a simple interpretation of CANAPE results. The second row shows grid cells that were selected to illustrate more complicated patterns than might have been expected. The bottom row shows grid cells that were selected to illustrate an unexpected result: these locations are significant in CANAPE yet contain none of the branches selected in RWiBaLD. The locations of these grid cells are shown in Fig 5A.

We mapped the spatial distribution of the three categories using the same 50 × 50 km grid cells as used in [1] in order to compare the RWiBaLD results to CANAPE results from the same data set. RWiBaLD provides details that extend and complement CANAPE. Locations identified by CANAPE as significant centers of endemism can be drilled into by looking at their constituent RWiBaLD branch classifications. For example, cells judged by CANAPE to be dominated by neo-endemism may contain one extremely neo-endemic clade (Fig 4A) or several moderately neo-endemic branches along with a meso-endemic branch (Fig 4E). Likewise, cells judged by CANAPE to be dominated by paleo-endemism may contain one extremely paleo-endemic clade (Fig 4B) or a moderately paleo-endemic branch along with a meso-endemic branch plus a moderately neo-endemic branch (Fig 4F). RWiBaLD is especially useful for examining cells classified by CANAPE as centers of mixed-endemism: some are truly mixtures of neo- and paleo-endemics (Fig 4C), some are only composed of meso-endemics (Fig 4D), some are complex mixtures of the three types of endemism (Figs 4G and 4H). Note that there can be cells identified as significant centers of endemism by CANAPE even when they contain none of the most highly endemic branches selected in step 2 of the RWiBaLD protocol (Fig 4I, 4J, 4K, and 4L). This underscores the differences between the two methods; CANAPE results are based on the ensemble of all branches present in the grid cell, not just those with the highest PE.

**Fig 5:**
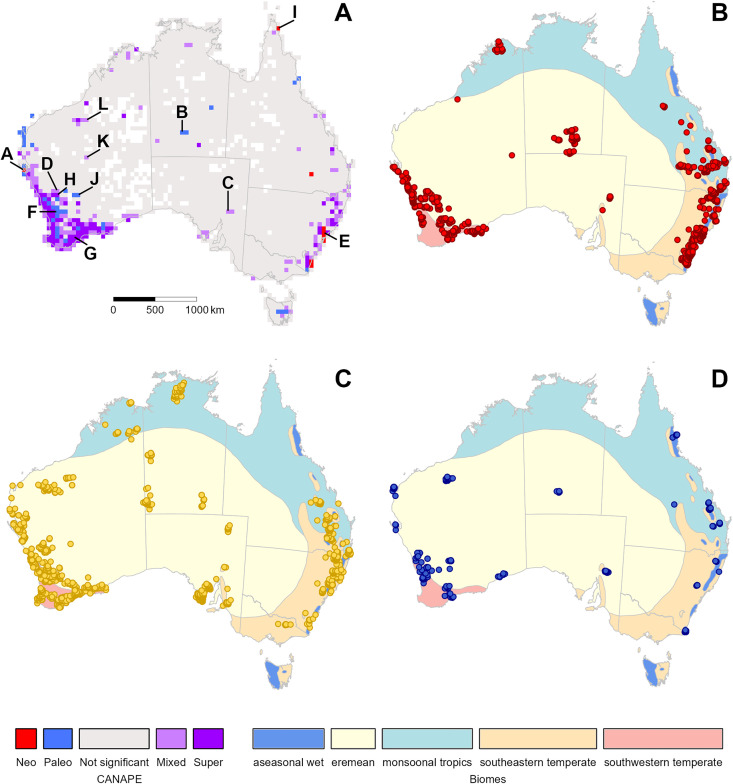
Results of RWiBaLD analysis in relation to CANAPE. **A.** CANAPE results using the data from Mishler et al. (2014); letters refer to the locations of selected grid cells shown in Fig 4. **B-D**. maps shaded with Australian biomes (adapted from Crisp et al. 2004) mapped with the distributional data of: (B) neo-endemic terminal branches (red), (C) meso-endemic terminal branches (yellow), and (D) paleo-endemic terminal branches (blue).

The geographic distribution of the branches of high PE identified in RWiBaLD broadly matches the centers of endemism found using CANAPE (Fig 5). However, examining the distribution of neo-endemic, meso-endemic, and paleo-endemic branches separately facilitates a more detailed understanding of patterns of endemism on the landscape, e.g., when looking for environmental, biological, or geological causes of endemism (e.g., Figs 5B-D). For example, our RWiBaLD results suggest neo-endemism to be more common than paleo-endemism in the relatively recent (3 Myr) central Australian arid zone biome [23]. In contrast, only paleo-endemic lineages occur in the wet tropics, the biome in Australia considered to be the oldest and most climatically consistent [24,25]. Complementary to the neo-endemism mapping largely to the youngest Australian biome, and paleo-endemism to the oldest, most of the meso-endemism branches map to the south-west and eastern temperate biomes – the biomes considered to be ‘middle-aged’ in Australian biogeographical history [26].

**Fig 6:**
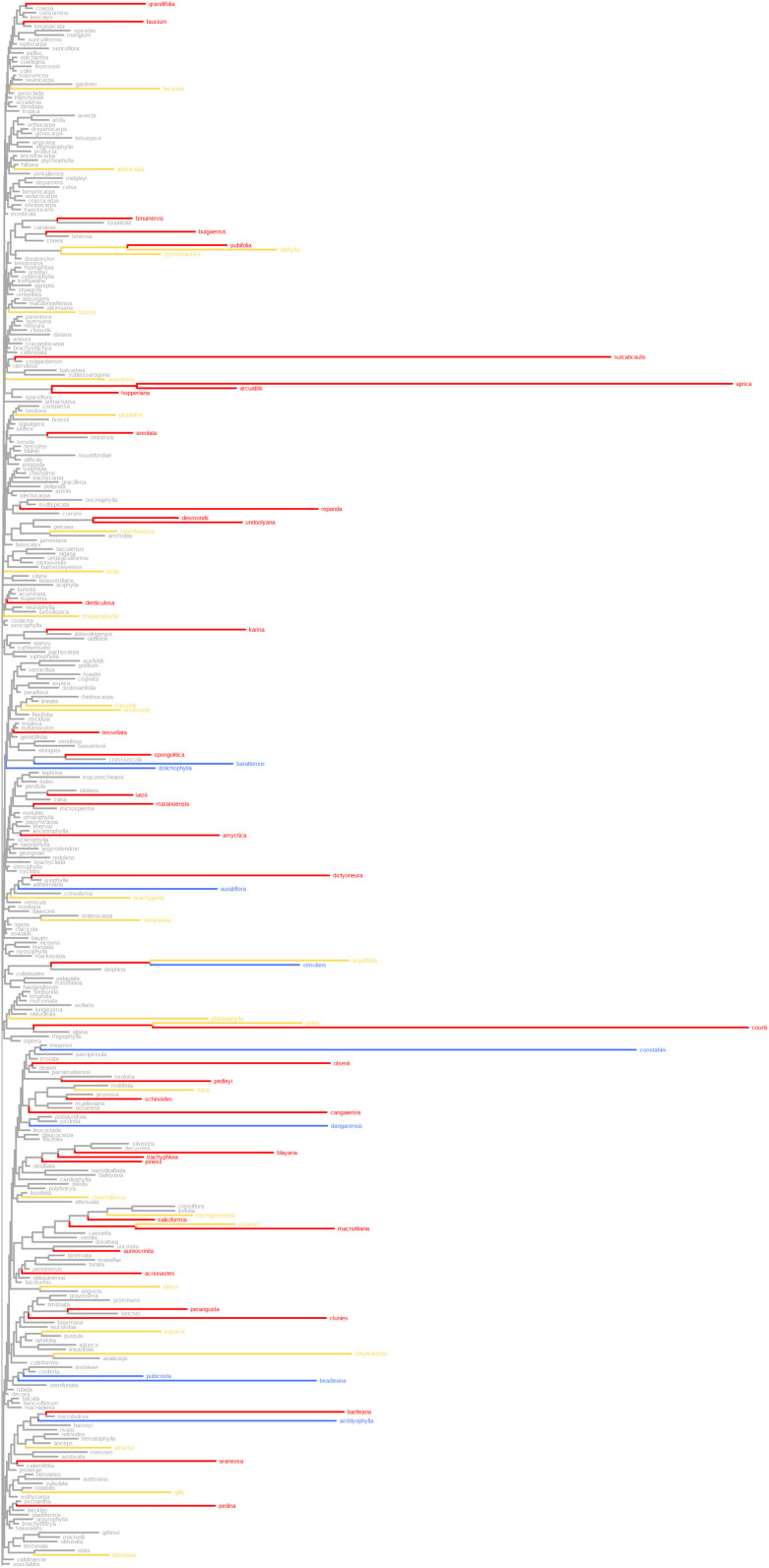
The three RWiBaLD categories mapped onto the *Acacia* range-weighted comparison tree, showing the top half of the tree. Blue = paleo-endemic branches, red = neo-endemic branches, yellow = meso-endemic branches.

**Fig 7:**
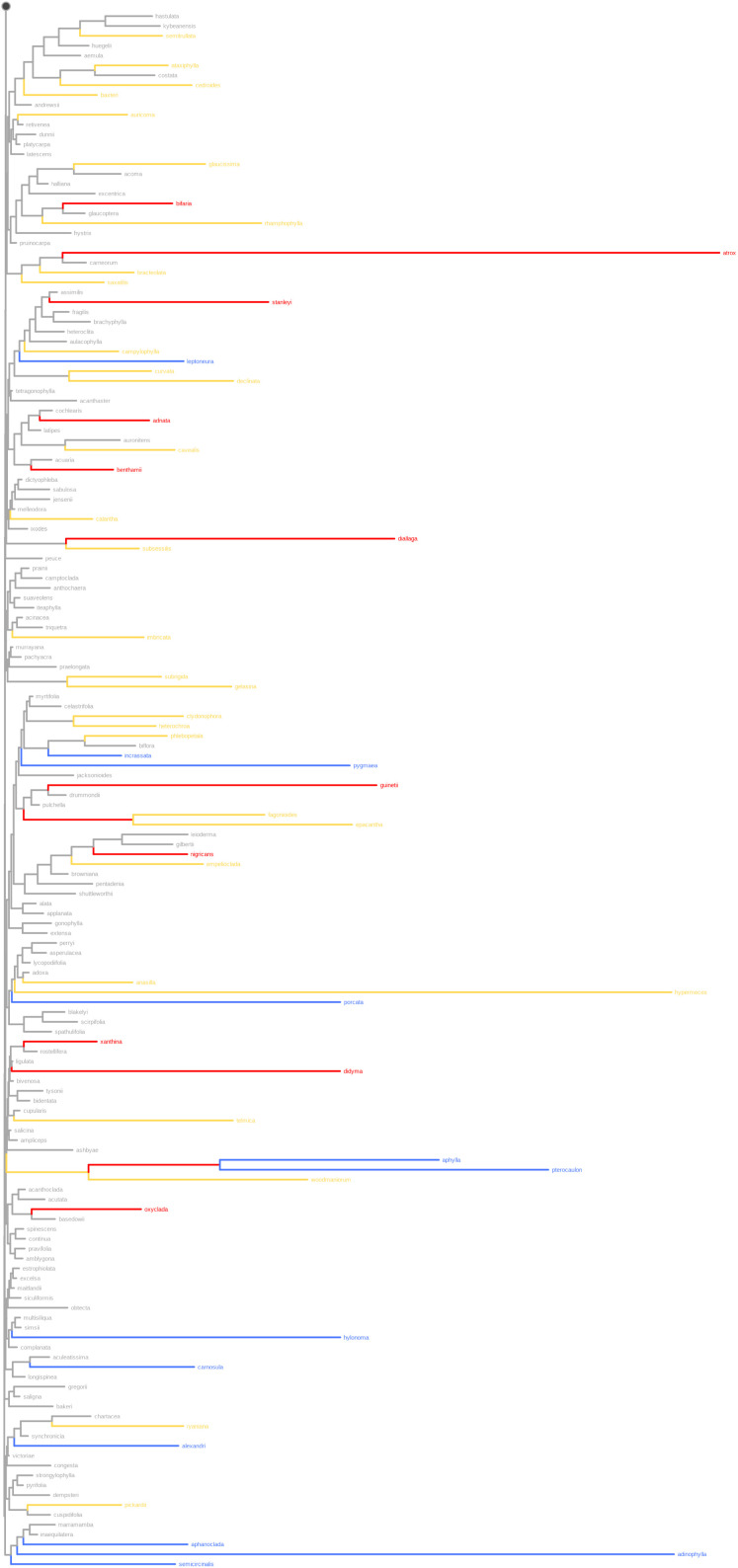
The three RWiBaLD categories mapped onto the *Acacia* range-weighted comparison tree, showing the bottom half of the tree. The black circle indicates where the subtree in Fig 6 connects with the topology shown here. Blue = paleo-endemic branches, red = neo-endemic branches, yellow = meso-endemic branches.

We illustrate mapping of the three categories of endemism onto the *Acacia* phylogeny in Figs 6 and 7. We used the range-weighted comparison tree, which starts out with equal branch lengths before range-weighting, so the branch lengths on that topology depend solely on how on how range-restricted the branch is (i.e., how few grid cells it occurs in). The colors show clearly how the longest branches get sorted into categories by RWiBaLD.

**Fig 8:**
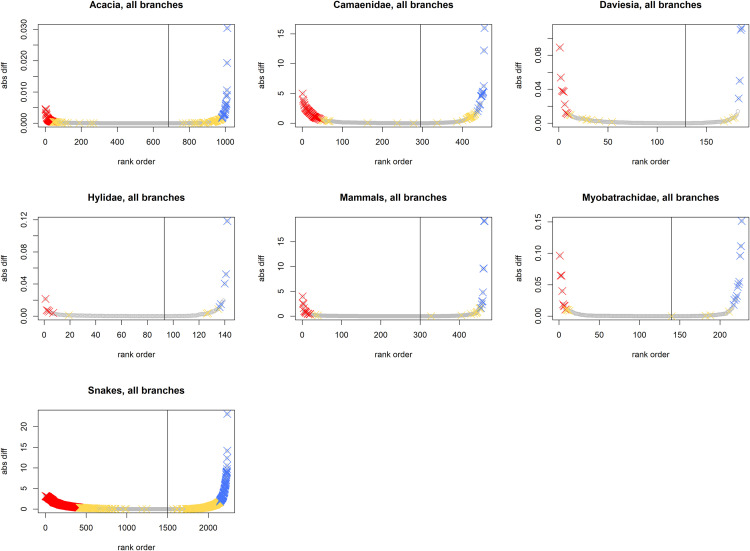
Illustrating the shape of the distribution of RWiBaLD scores for the seven comparison datasets, for all branches of the phylogeny. The Y-axis is the RWiBaLD score for each branch (the absolute difference is shown here); the X-axis is all branches ordered by RWiBaLD score. The branches of interest are colored by category (Red = neo; Yellow = meso; Blue = paleo); the remainder of branches are uncolored. The grey line indicates the inflection point, where RWiBaLD = 0. *Acacia* is not plotted here -- see Fig 2B for those results.

**Fig 9:**
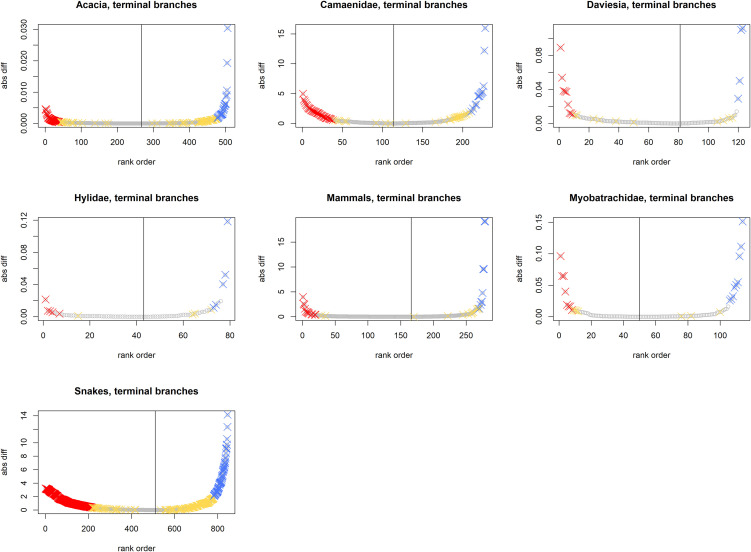
Illustrating the shape of the distribution of RWiBaLD scores for the seven comparison datasets, for only the terminal branches of the phylogeny. The Y-axis is the RWiBaLD score for each branch (the absolute difference is shown here); the X-axis is all branches ordered by RWiBaLD score. The branches of interest are colored by category (Red = neo; Yellow = meso; Blue = paleo); the remainder of branches are uncolored. The grey line indicates the inflection point, where RWiBaLD = 0. *Acacia* is not plotted here -- see Fig 2C for those results.

To explore the generality of the properties of RWiBaLD, we also examined seven additional previously published data sets [17,27] (Table 1). As shown in Table 2, Figs 8 and 9, results vary as might be expected from analyses on different organisms and at different scales, indicating that RWiBaLD should prove to be useful in discriminating among different evolutionary histories.

**Table 1:** Sources for the seven comparison datasets examined in order to look at the generality of the main results reported from the *Acacia* dataset.

Taxonomic group	Region	Source
*Acacia*	Australia	Mishler et al. (2014)
Passerines	Australia	Laity et al. (2015)
Camaenidae	Australia	Laity et al. (2015)
Daviesia	Australia	Laity et al. (2015)
Hylidae	Australia	Laity et al. (2015)
Mammals	Australia	Laity et al. (2015)
Myobatrachidae	Australia	Laity et al. (2015)
Snakes	South America	Guedes et al. (2018)

**Table 2:** Illustrating the number of the highly endemic branches of interest in the three categories (neo-endemic, meso-endemic, and paleo-endemic), found using the RWiBaLD protocol given in the paper, for the seven comparison datasets, plus the *Acacia* dataset studied in the main analyses.

RWiBaLD class	non-RW	Neo	Meso	Paleo
Acacia	873	55	59	21
Passerines	517	6	5	7
Camaenidae	370	42	29	14
Daviesia	161	9	12	4
Hylidae	128	5	4	5
Mammals	430	13	12	9
Myobatrachidae	201	8	8	10
Snakes	1140	199	304	48

## Discussion

### Factors affecting calculation of RWiBaLD

It should be noted that the RWoT used in RWiBaLD might be either a phylogram (where the branch lengths express inferred character change) or a chronogram (where the branch lengths express inferred elapsed time). Thus, there are two subtly different types of RWiBaLD, and one needs to be careful and explicit about selecting the phylogeny that is appropriate for a particular purpose. If the question involves the relative ages of branches, then the chronogram version of RWiBaLD is preferable. On the other hand, if the question involves the amount of genetic change along branches, e.g., fitting a common conservation goal of preserving genetic diversity, then the phylogram version of RWiBaLD is preferable. This distinction also applies to CANAPE.

Strictly speaking, paleo- and neo- are temporal concepts, so one should ideally use a chronogram to measure them. However, the terms paleo- and neo- have also been used to refer to long and short branches respectively on a phylogram without a time calibration [1]. These alternate ways of representing branch length are related of course, since most current methods of time-calibrating trees incorporate the branch lengths on the molecular phylogram (but not all methods [28]). Thus it is likely that results using a phylogram will be broadly correlated with those using a chronogram, but it is always worth checking for differences if both trees are available.

Note that most branches judged to be highly endemic with this protocol are terminal or close to the tips (Figs 6 and 7). This is because range sizes of branches are calculated as the union of the modern ranges of all descendants. With this approach, internal branches can be judged as highly endemic only if all their descendant branches are also range-restricted, something that becomes less likely as one moves deeper in the tree. If even one descendant is widespread then the deeper clade is not considered highly endemic, which is a limitation of this current approach to calculating PE for some purposes, especially regarding questions about deeper history.

Nonetheless, we think it is important to examine the RWiBaLD scores and endemism categories of all branch segments in the tree, terminal and deeper, in their own right. It is quite possible in theory that an ancestral branch segment was range-restricted and short in duration (thus *was* a neo-endemic branch), or range-restricted and long in duration (thus *was* a paleo-endemic branch), when a new split occurred. The new branches following the split have their own separate history. Thus it is conceptually possible that a branch that was young (at the time of splitting) and range-restricted (neo-endemic) gave rise to a branch that became old and range restricted (paleo-endemic), or vice-versa. To take a human analogy, someone could have a child when very young, yet the child might live to be very old. However, these possibilities are difficult to demonstrate in practice with current data and methodology; further methodological development is needed.

Focusing on current clade ranges to calculate PE, and thus only considering the range size in the present-day instantaneous time-slice, is appropriate for many purposes such as conservation assessment. However, this approach no doubt gives a poor estimate of the actual historical range size of deeper branches (or even earlier times in terminal branches). Thus, for some purposes, such as addressing questions in historical biogeography, it would be useful in the future to investigate methods for estimating past range sizes of deeper branches. For example, if a rich fossil history exists, one could make a range size estimate for a deeper branch that was independent of its descendant’s ranges. There are also methods in comparative genomics to infer previous effective population sizes that could be helpful in inferring past range sizes of branches [29,30]. Also promising are approaches that use occurrence data and environmental variables to provide estimates of ancestral niches and their associated projections in geographic space [31,32], and approaches designed for lineages that evolved via adaptive radiation that model how descendant taxa evolve to occupy portions of niches and trait space held by their ancestors [33,34].

The general motive for developing phylogenetic comparative methods arose from the need to move away from looking only at the current instantaneous time-slice, and to consider evolutionary time, taking into account both relationships among modern organisms and reconstructing changes along branches connecting them. While there are many biogeographic comparative methods available for reconstructing changes in range *location* [35], more attention in the future should be paid to reconstructing changes over time in range *size,* to facilitate more accurate studies of endemism through time and space.

### RWiBaLD in relation to BaLD

While this paper is focused on range-restricted branches, it is important to point out that contrasting branch lengths *without range-weighting* is also a useful approach and needs to be a part of the spatial phylogenetics toolbox. The measure BaLD introduced here, and the similar, previously published measure RPD [1], the ratio of branch lengths in a location on the original tree branch length to branch lengths on the comparison tree, are important metrics to compare with RWiBaLD and CANAPE. These metrics can help address questions about age of a biota in a region, centers of diversification, and refugia [2,3].

### Meso-endemism

To date, there has been a missing category in the study of endemism. The literature has focused on the ends of what is clearly a continuum of range-restricted branches with old, long branches (paleo-endemics) at one end, and new, short branches (neo-endemics) at the other. There clearly can be branches in the middle of that spectrum, range-restricted but not particularly long or short (Fig 1).

The term “meso-endemic” has previously been used for very different applications than those discussed here, e.g., in epidemiology it means a moderate amount of disease transmission in an area [36]. In biodiversity studies the term has been used to mean a medium-size distributional range [37]. We apply the term here to the previously undescribed case of branches in a phylogeny that are *highly* range-restricted but of medium *length*.

If paleo-endemics are lineages in the nursing home and neo-endemics are lineages in the nursery, then meso-endemics are lineages in mid-life crisis. The causes of endemicity are likely to be different for each category of endemism, and it is important to be able to distinguish among them in ecological, evolutionary, and biogeographic studies. Attention has been paid to finding explanations for neo-endemism and paleo-endemism since those terms were first coined [38–40]. Yet no attention has been paid to explanations for meso-endemics, largely because they could not be identified until now. RWiBaLD will enable such studies in the future.

### Empirical observations

The distribution of highly endemic branches in the *Acacia* example shows very few in the middle of the RWiBaLD distribution (Figs 2B and 3A). This is partly because deeper branches have larger range sizes, and thus their weighted branch length differences converge towards zero. When the data are plotted by increasing range weighted difference values, the deeper branches will plot near the inflection point between positive and negative differences. Thus the observation of fewer range-restricted branches in the middle of the distribution is partly an artifact of the deeper branches concentrated in the middle of the range of RWiBaLD values.

However, the same gap is observed when only terminal branches in Australian *Acacia* are visualized, as well as in the seven additional previously published data sets examined (Figs 8 and 9). In all of these data sets there are fewer highly endemic terminal branches that are near a medium length on the RWoT. This interesting phenomenon, where relatively few middle-aged, range-restricted lineages are observed, might be explained by what has been called the *taxon cycle* [41]. The basic idea being that branches tend to be range-restricted when young, achieve a broader range in their middle age, and shrink to a smaller range when old just before extinction. For many organisms, the probability of death is higher at younger stages of life and at older stages than it is in middle stages. To the extent that lineages resemble individuals, it would make sense that “middle-aged” lineages have a lower extinction rate. Causes for these patterns need to be pursued in the future; RWiBaLD will provide an explicit quantitative framework for facilitating the development of future studies testing hypotheses about taxon cycles.

The observation in the *Acacia* case study that neo-endemic branches outnumbered paleo-endemic branches also seems to be a frequent result. There were also more neo-endemic branches than paleo-endemic branches for four of the seven additional published data sets cited above (Table 2). More broadly, there appears to be a bias towards observing a majority of shorter branches on the original tree as well, as illustrated in Fig 2C. This apparent bias towards observing more short branches on phylogenies than long branches in the present time slice may be explained by the simple fact that newer lineages have had less time to go extinct than older ones. We see many of the short branches near the tips as preserved results of recent adaptive radiations, while many branches that diverged earlier are now gone.

While the above could explain why we observe more short than long branches in the RWiBaLD and BaLD distributions, there is a further skewing of observed data that also seems to be general: while fewer in number, the long branches depart farther positively from zero at the extremes than short branches do negatively (Fig 2B and 2C). The OT (or RWoT) can be any length longer than the CT (or RWcT) in the positive direction, but the difference is tightly bounded in the negative direction. In other words, there is more freedom to depart from zero in RWiBaLD and BaLD in the positive direction than in the negative.

Interestingly, the RWiBaLD and BaLD distributions are different in several ways. The RWiBaLD distribution shows a relatively sharp change in curvature near both the positive and negative ends (Fig 2B). On the other hand, the BaLD distribution shows no change in curvature at the negative end and a more gradual change in curvature at the positive end (Fig 2C). Furthermore, the highly endemic branches are scattered throughout the BaLD distribution in contrast to the pattern seen in RWiBaLD, where they are concentrated near the extremes (compare Figs 2B and 2C). These differences could be interpreted as indications that there are real-world processes affecting range-restricted branches particularly.

### Mapping RWiBaLD onto a phylogenetic tree

RWiBaLD allows us to classify a branch as neo-, paleo-, or meso-endemic. This in turn allows one to map types of endemism onto specific branches of a tree as traits (e.g., Figs 6 and 7). While this has been done for neo-endemism of terminal branches [40], there has heretofore not been a method to assign all endemism categories to any branch in a tree. This ability, plus the clear distinctions made by RWiBaLD between neo-, paleo-, and meso-endemism, will allow unprecedented studies of the causes of endemism in the future using standard comparative methods.

The RWiBaLD categories are a qualitative trait, and can be mapped as a categorical variable on the tree. We have described three main categories which we believe are suitable for most applications. However, one could of course divide the continuous distribution into further categories if appropriate, potentially using other threshold approaches. For example, branches in the meso-endemic category could be objectively parsed into two subcategories: those closer to neo-endemism and those closer to paleoendemism. Another possibility is that the non-endemic branches could be grouped into categories, allowing a global classification in which all branches have a designation, not just the endemic ones.

In addition, the RWiBaLD scores can be used as a quantitative trait to associate with branches of the tree. The two halves of the RWiBaLD distribution need to be mapped separately. Negative branch length differences represent a continuous measure of neo-endemism, while positive differences represent a continuous measure of paleo-endemism. One could then use standard comparative methods to compare RWiBaLD scores with quantitative traits hypothesized to affect endemism. An interesting issue is that while RWiBaLD scores are directly assigned to branches, as described above, methods such as independent contrasts use branch lengths on a tree to map quantitative traits scored for terminals onto deeper nodes, so one would need to consider which particular *facet* of the tree [14] is appropriate to use when mapping traits to compare with RWiBaLD scores. As mentioned in the first section of the discussion, there are two subtly different types of RWiBaLD, one based on the phylogram and one based on the chronogram. It would make sense for most purposes to use the same facet for comparing traits with RWiBaLD scores.

One could also attribute either the amount of PE or range size to a branch as quantitative traits for testing certain hypotheses. However, note that neither of those measures give the same information as RWiBaLD. As discussed above, branch length on the RWoT (PE) is biased towards paleo-endemism. The comparison tree uses mean branch length for the length of each branch *before* range-weighting, so directly reflects range size, but *after* range-weighing the RWcT branches differ in rank order of size from those in the RWoT. Using RWiBaLD, neo-endemic branches are longer on the RWcT than the RWoT, and vice-versa for paleoendemic branches.

Relative endemism (i.e., being range restricted [42]), functionally equivalent to previous approaches that used thresholds of range size to identify species/lineages of “geographic rarity” [43, 44], presumably can be caused by factors both intrinsic and extrinsic to the organism. Intrinsic factors might include breeding systems that limit cross-fertilization (e.g., self-compatibility), fruit/seed types that limit dispersal or establishment, or physiological preference for a rare soil type. Extrinsic factors might involve the physical environment, e.g., climate change such that a plant or animal’s physiology has become suboptimal, or the biotic environment, e.g., a plant’s major pollinator or disperser has disappeared, or a new competitor has pushed an animal out of most of its preferred environment. Tests of specific hypotheses of the causes of endemism in a particular group of organisms can now be tested using RWiBaLD in conjunction with appropriate phylogenetic comparative methods, examining functional traits (either qualitative or quantitative) that are thought to be causally related to endemism. Additionally, for ecological hypothesis testing it would be very helpful to add related metrics of rarity that take into account abundance [43,45], where the data permit.

### Visualizing RWiBaLD results on the landscape

When visualizing the spatial distribution of RWiBaLD results it is important to distinguish them from the results of CANAPE. Unlike CANAPE, RWiBaLD is not about providing a statistically significant summary classification for a particular location. It instead is about examining the individual distribution of highly endemic branches across locations. The three RWiBaLD categories can be separately mapped to show the geographic distribution of each (e.g., Fig 5 B-D). RWiBaLD categories could be shown on a map for individual grid cells, groups of grid cells, or larger subregions. One might focus particularly on locations that show significant concentration of PE in CANAPE to help understand what is driving statistically unusual patterns. Alternatively, one might have other criteria for selecting cells to examine, such as looking for environmental causes of endemism.

In addition to mapping the RWiBaLD categories, one could also map the two halves of the distribution of RWiBaLD scores as continuous measures of neo- and paleo-endemism (i.e., departure below and above the mean of the RWiBaLD distribution, respectively), using heat maps. This approach could be useful to address some questions, although it does not include a consideration of meso-endemism.

### RWiBaLD in relation to CANAPE

For most purposes, however, CANAPE results are the best suited to put on a map, followed by examining the RWiBaLD distribution within grid cells, where it can be seen that RWiBaLD is an important complement to CANAPE. This is particularly the case with the “mixed-endemism” category (including “super-endemism”) in CANAPE [1]. This category lumps together distinctly different situations. High PE locations that are not dominated by either paleo-endemism or neo-endemism might be truly mixed endemism in the sense of a mixture of paleo- and neo-endemic branches (e.g., Fig 4C). Alternatively, such locations might be dominated by meso-endemism, i.e., range-restricted branches of intermediate length, perhaps with no neo-endemic or paleo-endemic branches present at all (e.g., Fig 4D). Or, there might be a mixture of all three types of endemism (e.g., Figs 4G and 4H). Thus, RWiBaLD allows one to examine these mixed-endemism situations in greater detail, engendering greater understanding of what is occurring.

One important difference between CANAPE and RWiBaLD is that the former includes in its first step grid cells high in PE either on the RWoT or the RWcT, whereas the latter only includes in its first step branches with small range sizes (equivalent to PE on the RWcT). Because of the confounding of branch length and range size when measuring PE on the RWoT, CANAPE is relatively biased towards identifying paleo-endemism in comparison with RWiBaLD; e.g., there are more centers of paleoendemism than neo-endemism in the CANAPE map (Fig 5A), even though there are more branches judged by RWiBaLD to be neo-endemics (Fig 5B) than paleo-endemics (Fig 5C).

Locations that are identified by CANAPE as dominated by paleo-endemism or neo-endemism can also be more complicated than initially considered. CANAPE gives a single summary result for a location, and is responding to the overall signal across the whole phylogeny. In contrast, RWiBaLD considers the branches separately and thus enables a more detailed understanding of the situation. For example, RWiBaLD may sometimes reveal the presence of a neo-endemic branch in a grid cell otherwise dominated by paleo-endemic branches (e.g., Fig 4F). The unpacking of CANAPE results using RWiBaLD allows the detection of many different mixtures of branch length differences within a location, and greatly assists in developing possible causal explanations.

Thus, the two methods are complementary. CANAPE considers the PE contributed by all branches, to find statistically significant centers of endemism and classify them by their overall endemism type. In contrast, RWiBaLD is focused on the distribution of branches that have the highest PE. A location can be a significant center of endemism in CANAPE yet not contain any of the high PE branches identified in RWiBaLD. Conversely, a non-significant location in CANAPE can contain a branch with high PE. Thus the two methods are mutually supportive and should be used together to advance the study of endemism.

## Conclusion

RWiBaLD is a novel method that evaluates the contributions of each branch on a tree to total PE, allowing previously obscured phylogenetic and geographic patterns to emerge from data. RWiBaLD also enables hypothesis tests related to drivers of endemism, including new phylogenetic tests of traits associated with endemism, and new tests of spatial association of environmental factors with types of endemism. It can be applied to any group of organisms, including microbes. It will be useful to researchers studying eco-evolutionary processes, biogeographers assessing the spatial distribution of biodiversity, and conservation biologists setting priorities on the landscape.
